# Impact on Readmission Reduction Among Heart Failure Patients Using Digital Health Monitoring: Feasibility and Adoptability Study

**DOI:** 10.2196/13353

**Published:** 2019-11-15

**Authors:** Christopher Park, Emamuzo Otobo, Jennifer Ullman, Jason Rogers, Farah Fasihuddin, Shashank Garg, Sarthak Kakkar, Marni Goldstein, Sai Vishudhi Chandrasekhar, Sean Pinney, Ashish Atreja

**Affiliations:** 1 Icahn School of Medicine at Mount Sinai New York, NY United States

**Keywords:** heart failure, blood pressure, body weight, mHealth, remote consultation, patient care management, patient readmission, cell phone, mobile phone, blood pressure monitors, mobile apps

## Abstract

**Background:**

Heart failure (HF) is a condition that affects approximately 6.2 million people in the United States and has a 5-year mortality rate of approximately 42%. With the prevalence expected to exceed 8 million cases by 2030, projections estimate that total annual HF costs will increase to nearly US $70 billion. Recently, the advent of remote monitoring technology has significantly broadened the scope of the physician’s reach in chronic disease management.

**Objective:**

The goal of our program, named the Heart Health Program, was to examine the feasibility of using digital health monitoring in real-world home settings, ascertain patient adoption, and evaluate impact on 30-day readmission rate.

**Methods:**

A digital medicine software platform developed at Mount Sinai Health System, called RxUniverse, was used to prescribe a digital care pathway including the HealthPROMISE digital therapeutic and iHealth mobile apps to patients’ personal smartphones. Vital sign data, including blood pressure (BP) and weight, were collected through an ambulatory remote monitoring system that comprised a mobile app and complementary consumer-grade Bluetooth-connected smart devices (BP cuff and digital scale) that send data to the provider care teams. Care teams were alerted via a Web-based dashboard of abnormal patient BP and weight change readings, and further action was taken at the clinicians’ discretion. We used statistical analyses to determine risk factors associated with 30-day all-cause readmission.

**Results:**

Overall, the Heart Health Program included 58 patients admitted to the Mount Sinai Hospital for HF. The 30-day hospital readmission rate was 10% (6/58), compared with the national readmission rates of approximately 25% and the Mount Sinai Hospital’s average of approximately 23%. Single marital status (*P*=.06) and history of percutaneous coronary intervention (*P*=.08) were associated with readmission. Readmitted patients were also less likely to have been previously prescribed angiotensin-converting enzyme inhibitors or angiotensin II receptor blockers (*P*=.02). Notably, readmitted patients utilized the BP and weight monitors less than nonreadmitted patients, and patients aged younger than 70 years used the monitors more frequently on average than those aged over 70 years, though these trends did not reach statistical significance. The percentage of the 58 patients using the monitors at least once dropped from 83% (42/58) in the first week after discharge to 46% (23/58) in the fourth week.

**Conclusions:**

Given the increasing burden of HF, there is a need for an effective and sustainable remote monitoring system for HF patients following hospital discharge. We identified clinical and social factors as well as remote monitoring usage trends that identify targetable patient populations that could benefit most from integration of daily remote monitoring. In addition, we demonstrated that interventions driven by real-time vital sign data may greatly aid in reducing hospital readmissions and costs while improving patient outcomes.

## Introduction

### Background

Heart failure (HF) currently affects about 6.2 million people in the United States and has an approximate 5-year mortality rate of 42% [1,2]. With the incidence rate projected to rise by 46% to exceed 8 million cases by 2030, HF is an increasing public health concern [2]. Furthermore, although admission rates for HF have declined over the past two decades [3], readmission rates have not [1,4]; Centers for Medicare and Medicaid Services (CMS) data suggest that about 25% of HF patients are readmitted within 30 days after initial hospitalization, and 35% of these readmissions are because of HF [5]. About half of discharged HF patients are readmitted within 6 months post discharge. In 2012, the HF cost burden was estimated to be US $30 billion, and projections show that by 2030, total HF costs will increase 125% from 2012 to nearly US $70 billion, which averages to US $244 for each US adult [2].

Numerous studies have been aimed at understanding the greatest risk factors for HF readmission, and results are widely varied [6-13]. The American Heart Association reports that old age, African-American race, hypertension, diabetes mellitus (DM), and low socioeconomic status were associated with higher incidence of HF [1,2]. Other reported factors include nonclinical factors such as single marital status and Medicare/Medicaid status, and clinical factors such as renal failure, chronic obstructive pulmonary disease (COPD), and history of drug use [8,9,11,14-22]. Many studies have sought to build models that use these factors to predict readmission for HF patients [14,16,17,22-26]. However, these prediction schemes have struggled to universally reduce readmission rates for HF patients because of lack of evidence and inconsistencies in determining relevant factors [1,22,26].

Owing to the advent of remote monitoring technology, recent attempts to reduce readmission rates have begun to integrate modes by which physicians directly track their patients’ health statuses [27,28]. In addition to promoting a steadier communication between physician and patient, such protocols actively encourage patient participation in their own health, which has been shown to facilitate informed decision making and increase health literacy [29-31]. Studies utilizing remote monitoring for HF patients have shown varying but promising results; however, many of the reported solutions were done in research settings and required significant staff resources and costs associated with integration of telemonitoring [28,29,32,33].

### Objectives

In our quality improvement (QI) initiative, named the Heart Health Program, we developed a time-efficient and relatively cost-effective remote monitoring mobile device platform, prescribed from electronic health record (EHR)–connected platform, which helped to reduce hospital readmissions among HF patients. In addition to assessing readmission rates, we aimed to identify and analyze monitor usage and adherence trends in real-world home settings to inform future interventions to be conducted on larger scales.

## Methods

### Participants

The program included 60 patients who were admitted to the Mount Sinai Hospital for a diagnosis of acute HF. Under an approved QI protocol, eligible patients were approached around 2 days before discharge, and interested patients were enrolled in the Heart Health Program, a digital health monitoring initiative.

### Intervention Protocol

**Figure 1:**
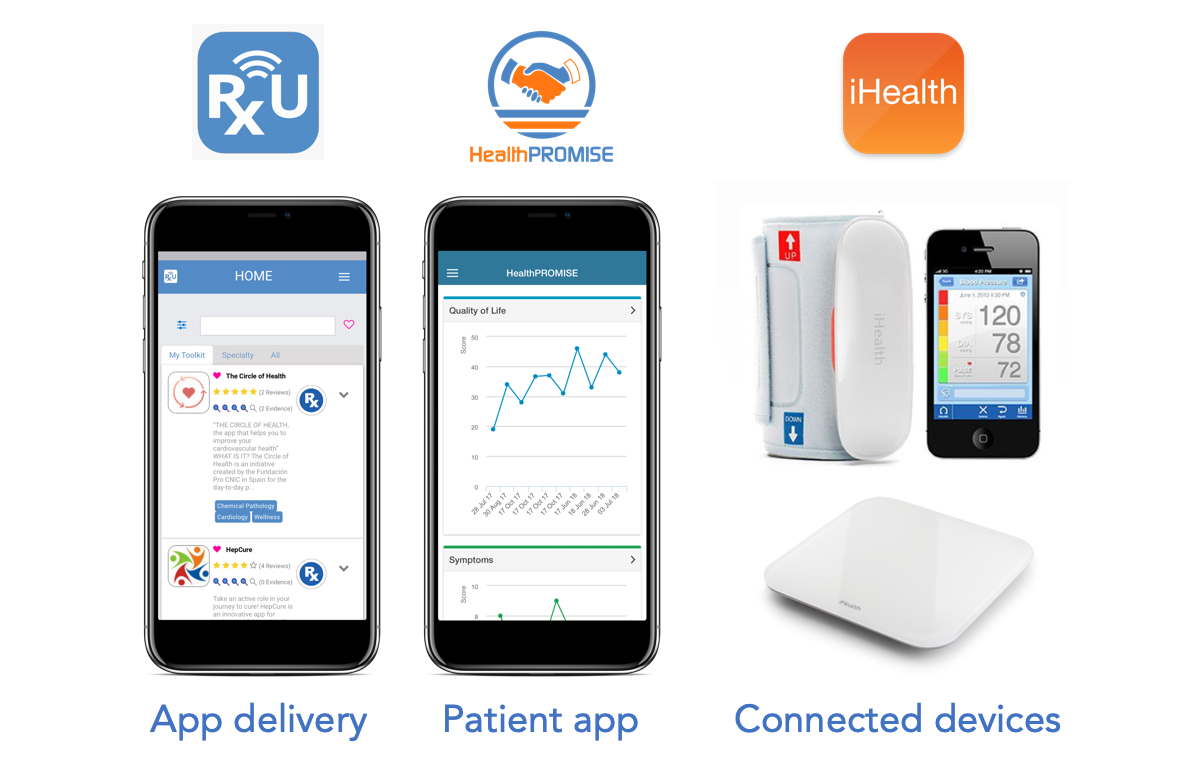
Digital care pathway “solution toolkit” for heart failure patients’ remote monitoring.

Our digital care pathway “solution toolkit” consisted of apps (HealthPROMISE digital therapeutic and iHealth mobile apps) that were prescribed using RxUniverse Digital Medicine platform (Rx.Health, Inc). RxUniverse is an EHR-integrated platform that allows digital prescription of automated care pathways including apps, digital therapeutics, education, and reminders directly to patients. This platform has been previously shown to have high usability and patient activation rate [34]. Patients were provided with HF education content, SMS or text reminders, and the ability to track their patient-reported outcomes (PROs) via preselected symptom checkboxes on HealthPROMISE digital therapeutic. In addition, each patient was provided with a complementary, consumer-grade Bluetooth-connected weight scale and blood pressure (BP) monitor that connected to the iHealth app installed on their own smartphone. Patients were instructed to measure their weight and BP each day. The total cost of equipment for each patient was US $110, paid for by the institution (Figure 1).

**Figure 2:**
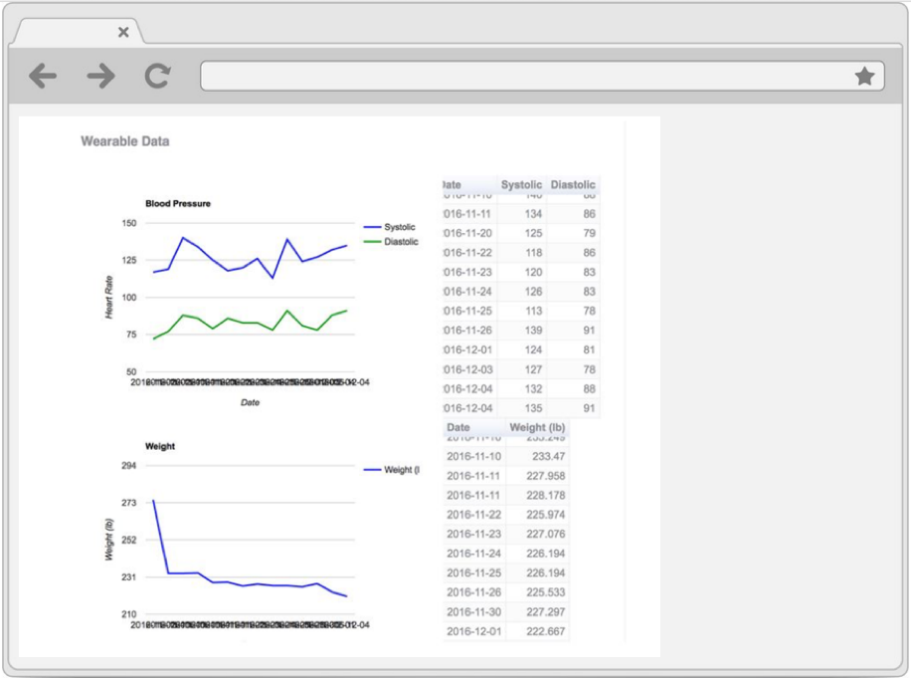
Screenshot of the electronic patient-reported outcomes data dashboard.

**Figure 3:**
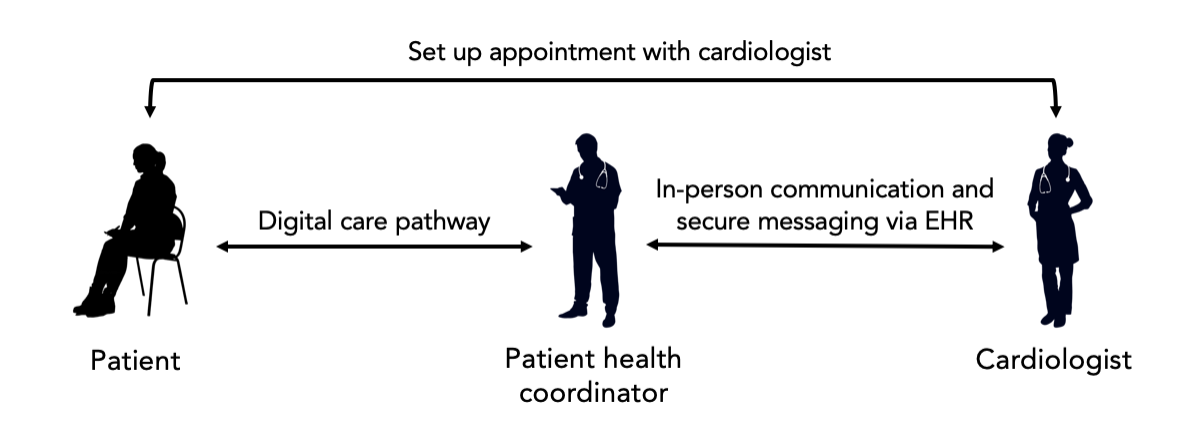
Intervention protocol pipeline and roles. EHR: electronic heath record.

Electronic PRO and Bluetooth scale and BP monitor data were sent to a Web-based dashboard (Figure 2), which was monitored daily by a patient health coordinator (coauthor, EO) who worked with the clinical team to assess symptoms and respond as needed. The clinical team comprised the nurse manager for the patient floor and a cardiologist. Any critical red-flag values, for example, a greater than 2-pound weight gain within 24 hours or greater than 5-pound weight gain within a week automatically alerted the physician and prompted the patient to seek medical attention. The alerted physician could then elect to have the patient contacted to set up an appointment with the cardiologist. Physicians were also able to contact patients based on individual judgment of changes in BP or weight. This pipeline is illustrated in Figure 3. This program was exempted by the institutional review board and classified as a QI study by the Department of Medicine’s QI Board.

### Dependent Variables

The primary outcome measured was 30-day all-cause readmission. In addition, usage of the monitors was defined as the number of times patients would use the weight and BP monitors per week following discharge. Usage for each monitor was calculated separately.

### Covariates

All patient data were pulled from EHRs, HealthPROMISE digital therapeutic, and Bluetooth-connected devices. Demographic variables included age, sex, race, and marital status. Clinical and social factors included insurance status; previous percutaneous coronary intervention (PCI); drug abuse; prescription of angiotensin-converting enzyme inhibitor (ACEI) or angiotensin II receptor blocker (ARB); prior HF; prior hospitalization within the last 12 or 6 months; and diagnoses of systolic HF, atrial fibrillation, COPD, depression, anxiety, cancer, coronary artery disease, HIV, DM, and anemia. Factors were selected based on a comprehensive literature search of factors correlated with HF readmission.

### Analysis

Readmission rates were compared with the reported Mount Sinai Hospital’s standard HF all-cause 30-day readmission rate as well as the national readmission rate. Patient characteristics were compared between the readmission and nonreadmission group using *t* tests and Fischer exact tests as appropriate. Finally, BP and weight monitor usage trends were qualitatively and quantitatively analyzed based on weekly usage percentage. All statistical tests are reported with the absolute *P* values.

## Results

### Patient Characteristics

A total of 60 patients were enrolled in the Heart Health demonstration program. Two patients dropped out because of personal reasons. Of the remaining 58 patients in the program, 33% (n=19) were female and 67% (n=39) were male, with a median age of 62 years (Table 1). Of the 58 patients, 57 were on Medicare or Medicaid. A majority of patients had been hospitalized in the prior 12 months before discharge (60%, n=36) and were single (55%, n=32).

### Readmission Statistics

Overall, there were 6 hospital readmissions (10%, 6/58) after 30 days, compared with the national 30-day readmission rate of 25%, which denotes a significant decrease (*P*=.06), and Mount Sinai Hospital’s readmission rate of 23%. As shown in Table 1, readmission was associated with single status (*P*=.06), a previous PCI (*P*=.08), and no prescription of ACEI or ARB (*P*=.02).

**Table 1:** Patient characteristics for 30-day readmitted and nonreadmitted patients.

Characteristics		Readmitted (n=6)	Not readmitted (n=52)	*P* value
Age (years), mean (SD)		63 (9.1)	58.7 (14.1)	.37
**Sex, n (%)**				.38
	Male	3 (50)	36 (69)	
	Female	3 (50)	16 (31)	
**Race, n (%)**				.46
	African-American	3 (50)	11 (21)	
	White	1 (17)	17 (33)	
	Other	2 (33)	23 (44)	
	Unknown	0 (0)	1 (2)	
**Marital status, n (%)**				.06
	Single	5 (83)	27 (52)	
	Not single	0 (0)	24 (46)	
	Unknown	1 (17)	1 (2)	
Systolic HF^a^, n (%)		5 (83)	37 (71)	>.99
Depression, n (%)		1 (17)	4 (8)	.43
Anxiety, n (%)		1 (17)	2 (4)	.28
Cancer, n (%)		2 (33)	8 (15)	.27
Coronary artery disease, n (%)		3 (50)	17 (33)	.41
History of drug abuse, n (%)		0 (0)	1 (2)	>.99
HIV, n (%)		1 (17)	0 (0)	.10
Atrial fibrillation, n (%)		1 (17)	17 (33)	.65
Chronic obstructive pulmonary disease, n (%)		0 (0)	4 (8)	>.99
Diabetes mellitus, n (%)		3 (50)	18 (35)	.66
Anemia, n (%)		4 (67)	16 (31)	.17
Percutaneous coronary intervention, n (%)		3 (50)	8 (15)	.08
Prior HF, n (%)		4 (67)	21 (40)	.39
Prior 12 months hospitalization, n (%)		5 (83)	31 (60)	.39
Prior 6 months hospitalization, n (%)		3 (50)	14 (27)	.34
Taking angiotensin-converting enzyme inhibitor/angiotensin II receptor blocker, n (%)		3 (50)	48 (92)	.02
Medicare/aid, n (%)		6 (100)	51 (98)	>.99

^a^HF: heart failure.

### Device Usage

**Figure 4:**
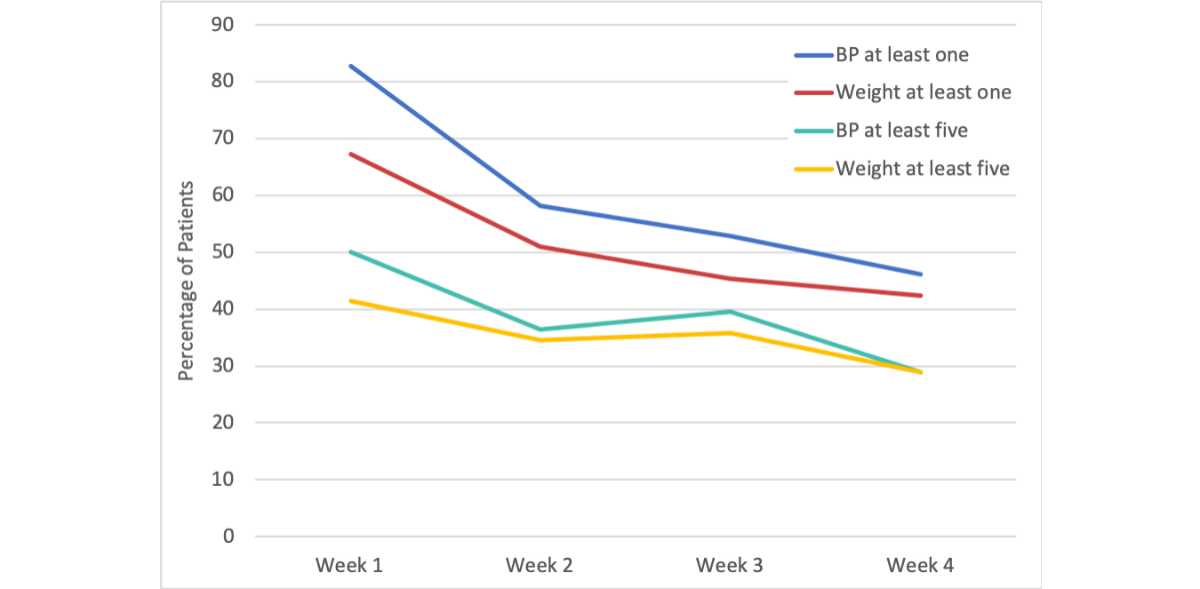
Absolute percentage of the total 58 patients with at least 1 or at least 5 blood pressure (BP) and weight checks each week following discharge.

The percentage of the total 58 patients using the monitors at least once dropped steadily from 83% (n=48) in the first week after discharge to 46% (n=27) in the fourth week (Figure 4; ie, in the fourth week following discharge, 46% of the original 58 patients, or 27 patients, were still using the BP monitor at least once). The percentage of patients using the monitors at least five times per week dropped from around 50% (n=29) to 30% (n=17) by the fourth week. Patients, on average, used the BP monitor more often than the weight scale. Device usage was not correlated with any patient characteristic or demographic factor.

Readmitted patients utilized the BP and weight monitors less frequently than nonreadmitted patients, and patients aged less than 70 years used the monitors more frequently on average than those aged more than 70 years. Furthermore, patients were more likely to use the monitors on weekdays compared with weekends. These trends, however, did not reach statistical significance.

## Discussion

### Overview

Readmission rates for HF patients have not decreased in the last few decades, and in fact, have risen, likely because of the decreased mortality and subsequent increased prevalence of HF patients [1]. Thus, strategies aimed at reducing readmissions are essential. The goal of this QI initiative was to assess feasibility (in part measured by readmission reduction and adherence rates) of using affordable digital health monitoring in real-world home settings, ascertain patient adoption trends, and evaluate impact on 30-day readmission rate as the primary outcome. We demonstrated that remote monitoring of BP and weight data via an app-based platform can be affordable, be adopted by patients irrespective of age, and reduce 30-day readmissions in real-world setting. We further identified usage trends that will be informative for future population health-wide implementations.

### Readmission Statistics

Our program’s readmission rate of 10% represented a significant decrease in 30-day readmission rate compared with both the Mount Sinai Hospital’s rate (23%) as well as the national rate (25%). Readmitted patients utilized the BP and weight monitors less frequently than nonreadmitted patients, and device usage was not correlated with any patient characteristic or demographic factor. These results support the published literature on the potential role of technology-aided remote care in HF patient management [27,30-32]. However, many of the published solutions were conducted in research settings and required invasive monitor placement, significant staff resources, or expensive equipment, which may not be necessary for all HF patients [31,32,34,35].

We were able to integrate an intervention protocol that reduced barriers to adherence on both the physician side and patient side by developing mobile apps that automated many parts of the workflow: PROs and monitor data were sent directly to a dashboard via mobile apps, and the patient health coordinator would only need to notify the physician of suspicious readings or symptoms. In addition, the RxUniverse platform would automatically notify patients to enter their symptoms on the app and measure their weight and BP. Thus, we were able to remove the need for telemonitoring, frequent check-ins for every patient, and the hassle that many patients may face in recording daily symptom and weight readings manually.

Besides physician monitoring of patient health status, interventions such as ours may also play a greater role in facilitating patient engagement with their own health [36]. For example, in receiving a weight scale, patients can be educated that quick increases in weight may reflect fluid retention because of high salt intake, which can exacerbate the HF symptoms. In this way, patients may be educated on their own condition, which has been shown to decrease mortality, morbidity, and costs associated with the disease [36-38].

### Device Usage

Decreased patient adherence to discharge protocols is one of the most prominent obstacles in HF patient management [39,40]. In our program, the vast majority of patients used the monitors in the week following discharge, and about half of the patients continued to measure their weight and BP at least once in the fourth week following discharge. The first week after discharge is the most vulnerable period for patients, and high engagement during that time was one of the important factors in reducing readmission rate. This drop in adherence from the first week onward may be attributable to numerous context-dependent factors, such as low motivation once patients know they are out of the “danger zone,” lack of long-term nursing or social support and/or living alone, and low technological proficiency [41]. Although some of these factors were beyond the scope of our program, these data provide insight into how intervention timing may be improved. As adherence falls after the first week, perhaps a front-loaded approach to increase patient engagement (via automated SMS text messages, direct interactive voice response calls, telemedicine and incentives when needed, etc) rather than an evenly spaced approach would increase and have a more long-lasting effect.

It is important to note that the number of patients taking BP and weight readings at least five times per week did not fall as drastically as the number of patients taking readings at least once per week. Furthermore, the majority of patients using the monitor at least five times in week 1 after discharge were the same as those in week 4. In other words, frequent monitor users are more likely to stay frequent users in the long run. Thus, patients that exhibit drastic decreases in monitor usage after the first week may represent a more specific targetable population.

In the past decade, remote monitoring, namely telemonitoring, has become a large field of interest in chronic disease management, and studies such as the Weight and Activity with Blood Pressure Monitoring System have described detailed protocols for such modalities [34]. In addition, studies that have implemented these monitoring solutions have showed great promise in reducing mortality and readmission [27]. However, many of the proposed workflows, in addition to the telemonitoring, require personnel-heavy intervention strategies, such as frequent phone calls and videoconferencing [27,29].

Many mobile apps have also been developed to help patients better manage HF, such as PatientConnect and HeartMapp [42]. Our app toolkit is similar to these apps in that it provides educational materials, care plans, reminders, in-app communication, and input options for PROs. In addition, our toolkit (1) integrates Bluetooth connectivity and automated updates of patient BP and weight data on the physician’s dashboard and (2) implements newly developed digital care pathways that can be delivered to patients without the need for mobile app downloads (eg, via SMS text messages). In our Heart Health Program, we reduced readmission by exclusively using remotely collected BP and weight readings, significantly reducing the cost and staff burden compared with other more resource-heavy interventions [29,32]. Coupled with the development of cheaper Bluetooth monitors and more effective risk stratification of patients who need monitors, our approach may be scaled as larger health systems look to expand digital health monitoring enterprise-wide to all HF patients. The Heart Health Program is now being expanded across multiple health systems through a nationwide digital transformation network in partnership with the American College of Cardiology.

### Limitations

Owing to our program being a demonstration program, our sample size was limited to 60 participants. Future research should use similar interventions on larger populations as well as different population demographics. Our sample size was also influenced by barriers to enrollment, including onboarding time, competition with other hospital initiatives and research trials, missed recruiting opportunities because of limited notice of discharge, language barriers, and smartphone ownership. Our program addressed these issues by enrolling HF patients around 2 days before expected discharge and adding a patient health coordinator to hospital rounds. Future research studies using device-dependent remote interventions on a larger scale could establish personnel with specific roles to address these issues and to maximize enrollment efficiency. Finally, consistent with real-world QI initiatives, specific physician-patient communications were not protocolized, and it was left to individual physicians to follow their best practices when patients’ alerts were seen. Additional work could be done to integrate a mobile app–enabled protocol where physicians may report their communications with patients to gain more granular data.

### Conclusions

Despite rigorous ongoing research, HF continues to contribute to increased health care costs and hospitalizations [1]. As technology advances, there is an increasing opportunity for a comprehensive, affordable, and personalized solution that can leverage existing technology to provide care to patients post discharge in real-world settings [31,43]. Recently, CMS approved reimbursement codes for remote monitoring and nonface-to-face chronic care disease management [44]. Diseases with increasing cost and health burdens such as HF could particularly benefit from such solutions. In our program, we demonstrated that interventions driven by real-time vital sign data may greatly aid in reducing hospital readmissions and costs, improving patient outcomes. Remote monitoring protocols such as ours both facilitate caregiver-patient interactions as well as engage the patient in his or her own health. Furthermore, remote monitoring programs have the potential to be scalable, especially when combined with digital medicine platforms integrated with EHRs. Future studies should seek to measure population health-wide impact on outcomes and revenue as digital health remote monitoring is expanded enterprise-wide.

## Abbreviations

ACEI: angiotensin-converting enzyme inhibitor
ARB: angiotensin receptor blocker
BP: blood pressure
CMS: Centers for Medicare and Medicaid Services
COPD: chronic obstructive pulmonary disease
DM: diabetes mellitus
EHR: electronic health record
HF: heart failure
PCI: percutaneous coronary intervention
PRO: patient-reported outcome
QI: quality improvement
